# A university system's approach to enhancing the educational mission of health science schools and institutions: the University of Texas Academy of Health Science Education

**DOI:** 10.3402/meo.v18i0.20540

**Published:** 2013-03-13

**Authors:** L. Maximilian Buja, Susan M. Cox, Steven A. Lieberman, Jonathan MacClements, Janet F. Williams, Robert M. Esterl, Kenneth I. Shine

**Affiliations:** 1 Department of Pathology and Laboratory Medicine, Medical School, The University of Texas Health Science Center at Houston, Houston, TX, USA; 2 Department of Obstetrics and Gynecology, The University of Texas Southwestern Medical Center, Dallas, TX, USA; 3 Division of Endocrinology, Department of Internal Medicine, The University of Texas Medical Branch at Galveston, Galveston, TX, USA; 4 Department of Family Medicine, The University of Texas Health Science Center at Tyler, Tyler, TX, USA; 5 Department of Pediatrics, School of Medicine, The University of Texas Health Science Center at San Antonio, San Antonio, TX, USA; 6 Department of Surgery and Transplant Center, School of Medicine, The University of Texas Health Science Center at San Antonio, San Antonio, TX, USA; 7 Health Affairs, The University of Texas System, Austin, TX, USA

**Keywords:** academy, consortium, faculty development, Health Science Education, Innovations Conference

## Abstract

**Background:**

The academy movement developed in the United States as an important approach to enhance the educational mission and facilitate the recognition and work of educators at medical schools and health science institutions.

**Objectives:**

Academies initially formed at individual medical schools. Educators and leaders in The University of Texas System (the UT System, UTS) recognized the academy movement as a means both to address special challenges and pursue opportunities for advancing the educational mission of academic health sciences institutions.

**Methods:**

The UTS academy process was started by the appointment of a Chancellor's Health Fellow for Education in 2004. Subsequently, the University of Texas Academy of Health Science Education (UTAHSE) was formed by bringing together esteemed faculty educators from the six UTS health science institutions.

**Results:**

Currently, the UTAHSE has 132 voting members who were selected through a rigorous, system-wide peer review and who represent multiple professional backgrounds and all six campuses. With support from the UTS, the UTAHSE has developed and sustained an annual Innovations in Health Science Education conference, a small grants program and an Innovations in Health Science Education Award, among other UTS health science educational activities. The UTAHSE represents one university system's innovative approach to enhancing its educational mission through multi- and interdisciplinary as well as inter-institutional collaboration.

**Conclusions:**

The UTAHSE is presented as a model for the development of other consortia-type academies that could involve several components of a university system or coalitions of several institutions.

Health science schools aspire to carry out the tripartite academic mission of teaching, research and service. Over the last 60 years, a number of relevant seminal events have impacted the mission of the health science institutions. These include the accelerated growth and importance of research activity catalyzed by funding from the National Institutes of Health, the National Science Foundation, and non-governmental scientific organizations; the accelerated growth and importance of the clinical enterprise following the advent of Medicare, Medicaid, and the contemporary fee-for-service payment system; the impact and influence of the pharmaceutical and biotechnology industries on academic institutions, and now, the evolving impact of seminal national health care legislation, the Patient Protection and Affordable Care Act (PPACA) of 2010. The contemporary environment is further complicated by a significant economic downturn, the Great Recession of 2008–10, the brief infusion of federal stimulus funding into the research enterprise followed by the loss of this temporary funding, the federal push for implementation of electronic health and medical records, and uncertainties for academic health institutions related to implementation of the PPACA, including Accountable Care Organizations, increase in Medicaid enrollment, etc. The long-term trends have placed intense emphasis on the research and clinical enterprises and have tended to diminish the educational mission and the educational role of the faculty. This development represents a paradox since education is the *sine qua non* for the existence of academic institutions. The current economic uncertainties impacting institutional funding are placing additional stress on the educational mission.

In response to concerns over the diminution of the educational mission and the role of medical education, the academy movement has developed in the medical schools in the USA over the last 10 years. Academies of medical educators represent formal organizations of academic teaching faculty who have demonstrated commitment and excellence in their contributions as educators and who serve specific needs in support of the educational mission of their institutions (1–5). Academies have the following characteristics: 1) a mission that advances and supports educators, 2) a membership composed of educators recognized for excellence and commitment, 3) a formal school-wide organizational structure with designated, often elected leadership, and 4) committed resources to support mission-related activities. Once launched, the academy movement spread rapidly. A 2008 survey identified that 21 medical schools had established academies and 33 schools were planning or considering academies (5).

Academies initially developed at medical schools constituted as components of individual universities. However, some medical schools and other health science schools are part of larger multi-campus institutions. In this situation, special challenges and additional opportunities exist for the academy movement. This is the case in Texas which provided the context to develop an innovation in the academy movement (6). This article presents the history and status of the response of the UTS to these challenges and opportunities.

## Medical and health science institutions of the University of Texas System

Texas has five major public educational systems, each of which is governed by a nine person Board of Regents appointed by the Governor. The University of Texas System is comprised of 15 major institutions, including nine general academic institutions and six health science institutions. Reporting to The University of Texas System Board of Regents, the UTS administration is led by a Chancellor and Vice Chancellors, including an Executive Vice Chancellor for Academic Affairs and an Executive Vice Chancellor for Health Affairs. A distinctive organizational feature of The University of Texas System is that the six health science institutions are free-standing entities that are not components of general academic campuses, as is the case with most medical schools in the USA. In chronological order of their establishment, the six health science institutions are: The University of Texas Medical Branch in Galveston (UTMB), The University of Texas MD Anderson Cancer Center in Houston (UTMDACC), The University of Texas Southwestern Medical Center at Dallas (UTSWMC), The University of Texas Health Science Center at San Antonio (UTHSCSA), The University of Texas Health Science Center at Houston (UTHSCH), and The University of Texas Health Science Center at Tyler (UTHSCT). These institutions have major research and clinical enterprises and also conduct multiple programs for the education of health care professionals and scientists at the undergraduate and postgraduate levels, including medical schools at four of the institutions (UTMB, UTSWMC, UTHSCSA, and UTHSCH). A variety of arrangements exist for linking these healthcare academic institutions with healthcare delivery systems.

## Chancellor's Health Fellows initiative

From the perspective of the UTS administration, overarching strategic considerations include the promotion of interactions among the component institutions to achieve efficiencies and reduce duplication while recognizing the history, culture, and unique aspects of the missions of the individual institutions. At the onset of the new millennium, the Vice Chancellor for Health Affairs and the Chancellor initiated a program to address strategic interactions among the health science campuses by the establishment of a Chancellor's Health Fellows program. The fellows were to be chosen from the faculty of the various health science institutions based on their experience and expertise in various fields. The fellows were to act as catalysts to promote collaborative activities and programs among the faculty of the health science campuses. In 2003, one of the authors (LMB) was appointed as the first Chancellor's Health Fellow designated as the Chancellor's Health Fellow for Education. Since the first appointment, the Chancellor's Health Fellow program has continued to evolve. Since the program began, there have been three to four health fellows appointed per year for a current total of 27 in diverse fields including education, nursing, public health, science and quality of care and patient safety, health care ethics and communication, health policy, clinical effectiveness, trauma and injury programs, health care reform and reimbursement, health information technology, disabilities, collaboration, and systems engineering.

Initial steps initiated and conducted by the Chancellor's Health Fellow in Education included visits to the educational constituency at the six health science campuses and the appointment of a Steering Committee with representatives from each of the campuses. The group proposed to organize a conference on Innovations in Health Science Education which was held in October 2004. At this conference, the group proposed the formation of The University of Texas Academy of Health Science Education (UTAHSE) of the UTS.

## The University of Texas Academy of Health Science Education

**Fig. 1:**
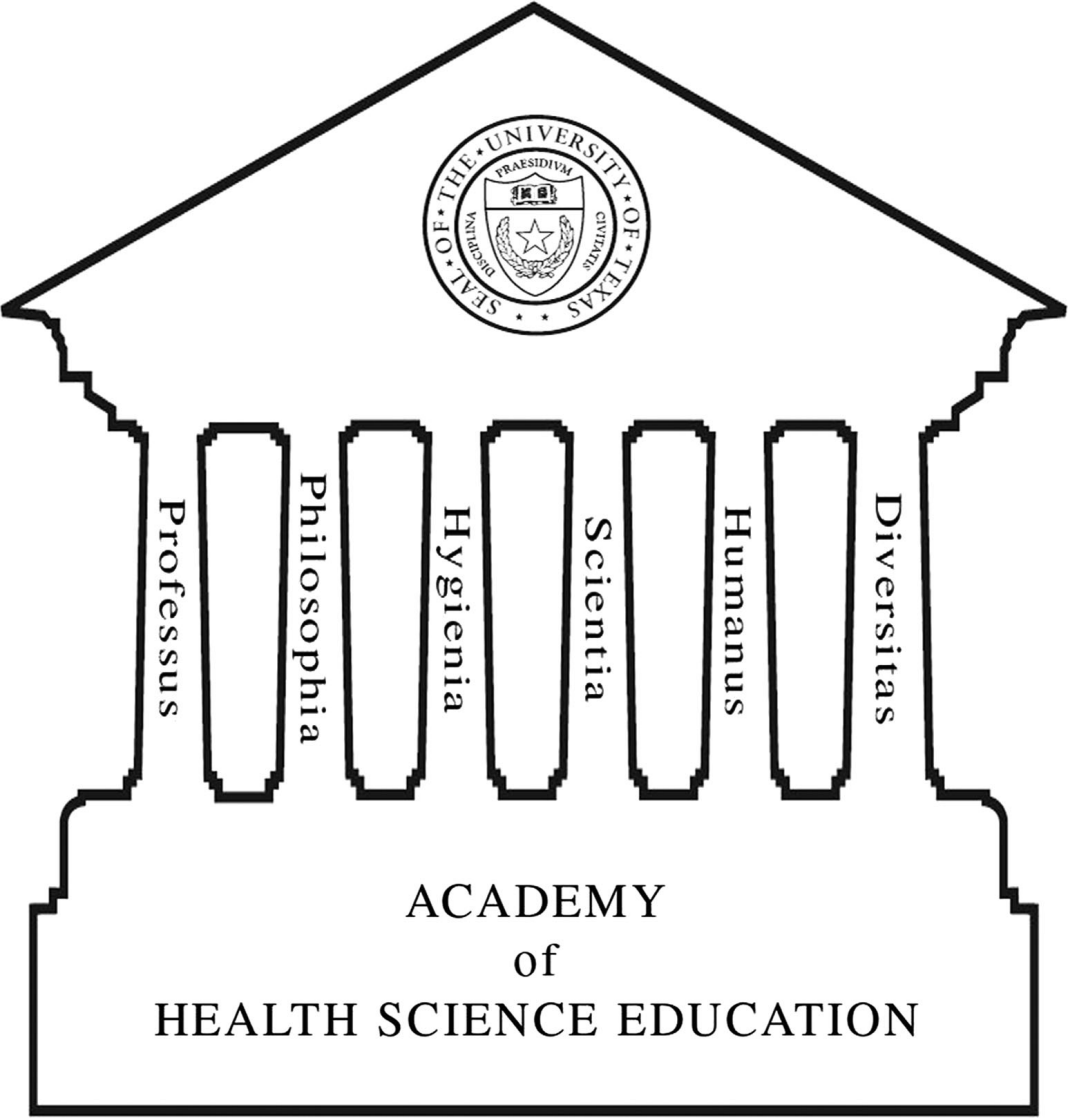
Logo of the University of Texas Academy of Health Science Education depicting a temple of scholarship bolstered by the six pillars representing the guiding principles of the Academy.

With the support of the Vice Chancellor and the presidents of the six health science institutions, the UTAHSE was formed with the appointment of 13 founding members selected by the presidents of the six health science institutions. These individuals had diverse backgrounds in medicine, nursing, and biomedical science. Discussions led to consensus on a Mission Statement and key guiding principles. The stated mission of the UTAHSE is to foster excellence in education in the health sciences by recognition of outstanding educators and advancement of knowledge and innovation in the field of education. The UTAHSE is built around six campuses and six pillars representing six guiding principles (Fig. 1). These are: 1) Professus – a person who professes something; a teacher, 2) Philosophia – love of wisdom and knowledge, 3) Hygienia – the science of health and its maintenance, 4) Scientia – systematized knowledge derived from observation, study, and experimentation, 5) Humanus – a system or way of thought or action concerned with the interests and ideals of people, and 6) Diversitas – collegial interaction among all. The stated goals of the UTAHSE are: 1) reward outstanding educators for their exceptional contributions, 2) support faculty development for education, 3) promote the academic advancement of teachers in the health sciences, 4) encourage development and implementation of innovative educational projects, including collaboration between disciplines and institutions, 5) promote curriculum design and reform, and 6) foster educational scholarship and research of teaching faculty and provide financial assistance for new and innovative educational projects. The scope was meant to include educational efforts related to medical students and other undergraduate health science students and curricula as well as graduate and postgraduate education including graduate medical education for clinical residents and fellows.

The founding members created bylaws and policies and procedures for the Academy. Regarding organization and governance, the bylaws established the following: 1) Executive Board, consisting of the four officers—President, Past-President, President-Elect, and Secretary-Treasurer; 2) Standing Committees – Membership, Faculty Development and Educational Program, and Awards; and 3) Advisory Board comprised of the officers and chairs of the standing committees. A UTAHSE web site was established to promote communication regarding the Academy (http://www.utsystem.edu/academy/hse/).

## Membership in the UTAHSE

From the outset, it was agreed that membership was to be honorific based on achievement coupled with a commitment to service to the mission of the Academy. Input from the institutional presidents reinforced the selective nature of the election to membership. The bylaws allowed for the election of 24 new members for the first 2 years and no more than 12 new members per year thereafter.

Eligible members in the Academy must be outstanding teachers in the University of Texas health science system. A goal of the Academy is to have diverse membership representing the many disciplines in health science education. For eligibility for membership in the Academy, educational excellence is characterized broadly to include, but not necessarily to be limited to: 1) direct teaching, 2) curriculum development, 3) counseling and mentorship, 4) educational administration and leadership, and 5) educational scholarship and research.

The application for membership submitted by an officially nominated candidate is to include: 1) a statement of commitment including new or continuing contribution to the UT Academy and the University of Texas as an Academy member; 2) a highlighted summary of the candidate's outstanding educational contributions; 3) a personal statement which includes: (a) a discussion of the candidate's educational philosophy, (b) a discussion of the candidate's professional development efforts in education, (c) a discussion of the candidate's intended contributions to the Academy; 4) a complete and current curriculum vitae; and 5) five letters of support: one of which will be the official letter of nomination, two of which should come from peers including faculty at the home or other institution, and two of which should come from learners who benefited from the educational expertise of the candidate.

The annual review and selection of new members of the Academy has evolved since the beginning of the Academy. Initially the process was performed by the entire membership acting as a committee of the whole. Currently, the Membership Committee chair assigns the application portfolios of the candidates for review by three primary reviewers. The full Committee then reviews the portfolios and initial average scores of the candidates, and then after full discussion, makes a final determination of a slate of candidates to present to the general membership for election into the Academy. The selection process focuses on the following criteria: 1) breadth and variety of the educational contributions, 2) quantity of the educational contributions, 3) quality of the educational contributions, 4) effectiveness of the educational contributions measured by outcomes, 5) reputation as an educator, 6) length of time at health science center(s) with active involvement in educational effort, 7) percentage of professional activity devoted to the educational effort, and 8) commitment to the UTAHSE.

Currently the UTAHSE has 132 regular members. Physicians (MDs) and medical schools have a strong representation. However, the entire membership now comprises multiple health science professionals with representation from all six of the health science institutions generally reflecting the different composition of health professional units at these institutions (Table 1). Thus, progress has been made in achieving the stated goal of a diverse membership for the Academy.

**Table 1:** Members of the Academy of Health Science Education for the years 2005–13 according to Health Science Institution and primary degree related to professional field

Institution	MD	RN/MSN/PhD/or equivalent	PhD or equivalent in basic or applied science	PhD or equivalent in allied health	DDS	Total
UTMB	14	3	8	3	0	28
UTMDACC	12	0	11	0	0	23
UTSWMC	13	0	7	1	0	21
UTHSCSA	9	0	12	3	0	24
UTHSCH	13	8	10	0	3	34
UTHSCT	1	1	0	0	0	2
Total	52	10	38	7	3	132

Note: In addition to regular members, there have been 11 members elected to the UTAHSE who are now Emeritus Members, either because of retirement or departure for a UT institution. These include three from UTMB, two from UTMDACC, three from UTHSCSA, one from UTSWMC, and two from UTHSCH, and represent five MDs and six PhDs. The regular membership of the UTAHSE includes two individuals who are retired from full time employment but remain active in the UTAHSE. There is one honorary member of the UTAHSE (Dr. Kenneth Shine). UTMB=University of Texas Medical Branch at Galveston; UTMDACC=University of Texas M. D. Anderson Cancer Center; UTSWMC=University of Texas Southwestern Medical Center at Dallas; UTHSCSA=University of Texas Health Science Center at San Antonio: UTHSCH=University of Texas Health Science Center at Houston; UTHSCT=University of Texas Health Science Center at Tyler.

The composition of UTAHSE members from the six health institutions reflects the differences in scope of each of the health institutions. Three institutions do not have RN members because those institutions do not have nursing schools. There are PhD members from most of the institutions who are engaged in graduate science education, health professional education, or both. The relative differences in PhDs and health professional degree members reflect the outcome of the membership selection process including local nominations and selection by the UTAHSE Membership Committee.

Another goal of the UTAHSE has been to promote the establishment of local academies at the six UT health science campuses as well as interactions among the local academies and the UTS Academy. The following local academies are now in operation (date of founding): Academy of Master Teachers at UTMB 2006, Southwestern Academy of Teachers (SWAT) at UTSWMC (2006), Academy of Master Teachers at UTHSCHSA (2008), Academy of Medical Educators at the University of Texas Medical School at Houston (2010), and Academy of Health Science Educators at UTMDACC (2010). The UTAHSE and the local academies have a mutually supportive relationship. However, membership in the UTAHSE does not require prior membership in a local academy or vice versa.

## Activities of UTAHSE

The major activities of the UTAHSE include an annual Innovations in Health Science Conference, Outstanding Educational Innovation Awards, and a small grants program. The innovations conference has been a major annual highlight for the Academy. The conferences have featured important educational themes and have included presentations by nationally known educational leaders from outside of Texas as well as Academy members from the UT campuses. The themes of the conferences are presented in Table 2. Most of the conferences have taken place on ‘neutral ground’ in Austin, Texas. The lead in organizing the conference is currently taken by the president-elect. Attendance is not limited to UT faculty and is open to any interested individuals. Attendance has grown from 66 registrants for the first conference to over 150 registrants for the more recent conferences.

**Table 2:** Innovations in Health Science Education Conferences

2004	Innovations in medical education and curriculum development
2005	Competencies and professionalism in medical education
2006	Faculty development and the Academy movement
2007	Focus on communication skills and response to medical errors
2008	Health care quality and interprofessional learning
2009	Hurricane Ike – no fall conference – conference moved to the early spring
2010	Finding the time, finding the money, improving the efficiency and quality of health care education
2011	Interprofessional health science education: the innovation imperative
2012	Assessing students in health science education – how we teach; how they learn
2013	Sustaining a highly productive academic environment: raising the priority for mentoring

The Academy solicits nominations for new educational projects to be supported by a small grants program. Additionally, applications are solicited from faculty for Outstanding Educational Innovations Awards based on ongoing or completed projects with demonstrable outcomes. The Awards Committee selects up to six projects for small grant awards in the range of $5,000 each. The Awards Committee also selects first, second, and third place for the Outstanding Educational Innovations awardees based on written submissions and presentations at the annual innovations conference. The awardees receive recognition and honoraria in the range of $1,000–3,000. The small grants and awards have involved a range of projects, including faculty development; assessment of professionalism; development of communication skills; promotion of proactive learning; use of standardized patients, interdisciplinary education, public health preparedness, interface with the electronic health record and learning modules in a variety of areas, including radiology, anatomy, statistics; and various procedures. More detail is provided at the UTAHSE web site (http://www.utsystem.edu/academy/hse/). Funding for an annual budget in support of the Innovations Conference, the Outstanding Educational Innovations Awards, and the small grants program has been provided by The University of Texas System Chancellor's Office.

## Outcomes of the UTAHSE

Through work of planning committees and the conduct of the annual innovations conference, the UTAHSE has served as a catalyst to foster increased collegiality and multiple layers of interaction and coordination among members from the six campuses. Some examples of specific outcomes are as follows. As a result of discussions at one of the early innovations conferences, colleagues from UTSWMC provided curricular resources for the establishment of a training program for medical residents at UTHSCT. One of the small grants went to support the establishment of an online series of teaching cases in surgery for medical students at UTSWMC. Student performance showed an average increase of 10 percentile in the 6 years since initiation of the program compared to the previous several years. At a practical level, relationships established among UTAHSE members facilitated the smooth and rapid success in placement of medical residents from UTMB at other UTS institutions in the aftermath of Hurricane Ike. Members of the UTAHSE credit the UTAHSE as a major force in advancing the goal of medical education throughout the UTS.

## Additional related educational efforts of The University of Texas System

The positive outcome of the establishment of the UTAHSE contributed to the launch of additional related educational initiatives by UTS. In 2008, the UTS Executive Vice Chancellor for Health Affairs established an Innovations in Health Science Educational Program reporting directly to this office and charged with working on specific projects to advance health science education. In 2008, the University of Texas Board of Regents allocated $5 million dollars for the work of this group.

A request for proposals was allowed for major projects to achieve innovations in medical education. After review and grading of the proposals by a panel of experienced educators, grants have been awarded to six groups of investigators at a level of funding of $100,000 a year for 3 years each. Four of the projects have co-investigators from multiple campuses. Currently all projects are progressing well and are on schedule. Funded projects range from technology-based instruction and faculty development to community service learning and palliative care education.

A conversation among the presidents of the 15 UTS institutions led to the idea of a thorough reformulation of physician education from the end of high school through to the receipt of the MD degree. The Transformation in Medical Education (TIME) initiative has been launched with the full support of Vice Chancellor for Health Affairs, Dr. Kenneth Shine, and Chancellor, Dr. Francisco Cigarroa, and the moral and financial support of the UT Board of Regents. The goal of this initiative is to build upon earlier efforts to increase the effectiveness and relevance of physician education while shortening its duration (7). To this end, the UTS academic and health campuses have formed partnerships of two to five schools to develop, implement, and assess pilot programs of fully revamped premedical and medical education with an eye toward scalability beyond the pilot phase. The strong and collaborative relationships established among leading UTS medical educators through the UTAHSE have greatly facilitated the progress on this ambitious project.

Other ongoing components of the UT Innovations in Health Science Education are a program in global health and a Texas Learning Object Repository which is currently under development.

## Recognition of health science educators

The University of Texas System Board of Regents has received periodic reports of the activities of the UTAHSE and the other educational projects discussed above. In the summer of 2010, members of The University of Texas System Board of Regents hosted a reception and dinner for members of the UTAHSE. A highlight of the event was the presentation of medallions engraved with the logo of the Academy to each of the members. These medallions are intended for use as part of the academic regalia at graduation ceremonies and other appropriate events.

The University of Texas System Board of Regents has approved the award of the title, Distinguished Teaching Professor, for distinguished academicians and educators on faculty at any of the University of Texas institutions. The Executive Vice Chancellor for Health Affairs has promoted the opportunity for members of the UTAHSE and/or members of local academies to be nominated for this academic title. Award of the title requires review and approval by the President of the candidate's institution and the Executive Vice Chancellor for Health Affairs.

## Summary and conclusions

The UTAHSE has served an important function in the large multi-campus University of Texas System to recognize the importance of the educational mission and the contributions of health science educators and to promote interdisciplinary and collaborative innovations in health science education. The UTAHSE can serve as a model for the development of other consortia-type academies involving components of university systems or coalitions of several individual institutions. The UTAHSE continues to pursue the overarching goals of promoting excellence in our faculty and students in order to advance medical and health science knowledge and transmit that knowledge for the benefit of the health of our citizens.
